# Methyl eugenol increases neural microcirculation to promote nerve repair by regulating endothelial cell and Schwann cell communication via Ptn/Sdc4

**DOI:** 10.1007/s13659-026-00617-z

**Published:** 2026-10-02

**Authors:** Zifeng Zhuang, Shaozi Lin, Yixuan Li, Xiaokang Xie, Hongling Chen, Yuting Hao, Lan Lin, Guoping Zhao, Di Zhang

**Affiliations:** 1 College of Traditional Chinese Medicine, Jinan University, Guangzhou, China; 2 Guangzhou Medical University Affiliated Traditional Chinese Medicine Hospital, Guangzhou, China

**Keywords:** Methyl eugenol, Schwann cells, Endothelial cells, Ptn, Sdc4

## Abstract

Neuromicrocirculatory disorders often accompany nerve injury. Improving neural microcirculation can promote the repair of injured nerves. Methyl eugenol is a major bioactive component of *Asarum heterotropoide*s and has been reported to exert analgesic effects in chronic pain. This study aimed to demonstrate that methyl eugenol can enhance neural microcirculation and thereby promote nerve repair. A combination of single-cell sequencing and mRNA sequencing revealed that pleiotrophin (Ptn)/syndecan-4 (Sdc4) is vital for coupling between endothelial cells and Schwann cells. A total of 32 rats were randomly assigned to four groups, namely, the Sham, chronic constriction injury (CCI) 7d, CCI 14d, and CCI 21d groups, to assess whether endothelial Schwann cell communication is diminished following CCI. After an endothelial cell–Schwann cell coculture system was established, methyl eugenol, Ptn siRNA, and Sdc4 siRNA were administered. In a separate experiment, 32 SD rats were randomly divided into four experimental groups: the Sham, CCI, ibuprofen, and methyl eugenol groups. CellChat analysis revealed that nerve injury reduced the degree of communication between endothelial cells and Schwann cells, especially communication via Ptn/Sdc4. CCI disrupted neural microcirculation, lowered Ptn levels, and increased Sdc4 expression. In cell experiments, methyl eugenol promoted rat aortic endothelial cells tube formation and increased the release of Ptn, which acted on Sdc4 in rat Schwann cells (RSC96) to promote migration, but did not directly promote RSC96 cell migration in the absence of endothelial cell–derived factors. Treatment with methyl eugenol increased neural microcirculation to promote nerve repair in rats subjected to CCI. In addition, methyl eugenol further increased Ptn levels. CCI led to neural microcirculatory dysfunction and nerve injury. Methyl eugenol promote nerve repair by enhancing neural microcirculation through regulation of endothelial cell and Schwann cell communication via Ptn/Sdc4.

**Graphical abstract:**

**Figure:**
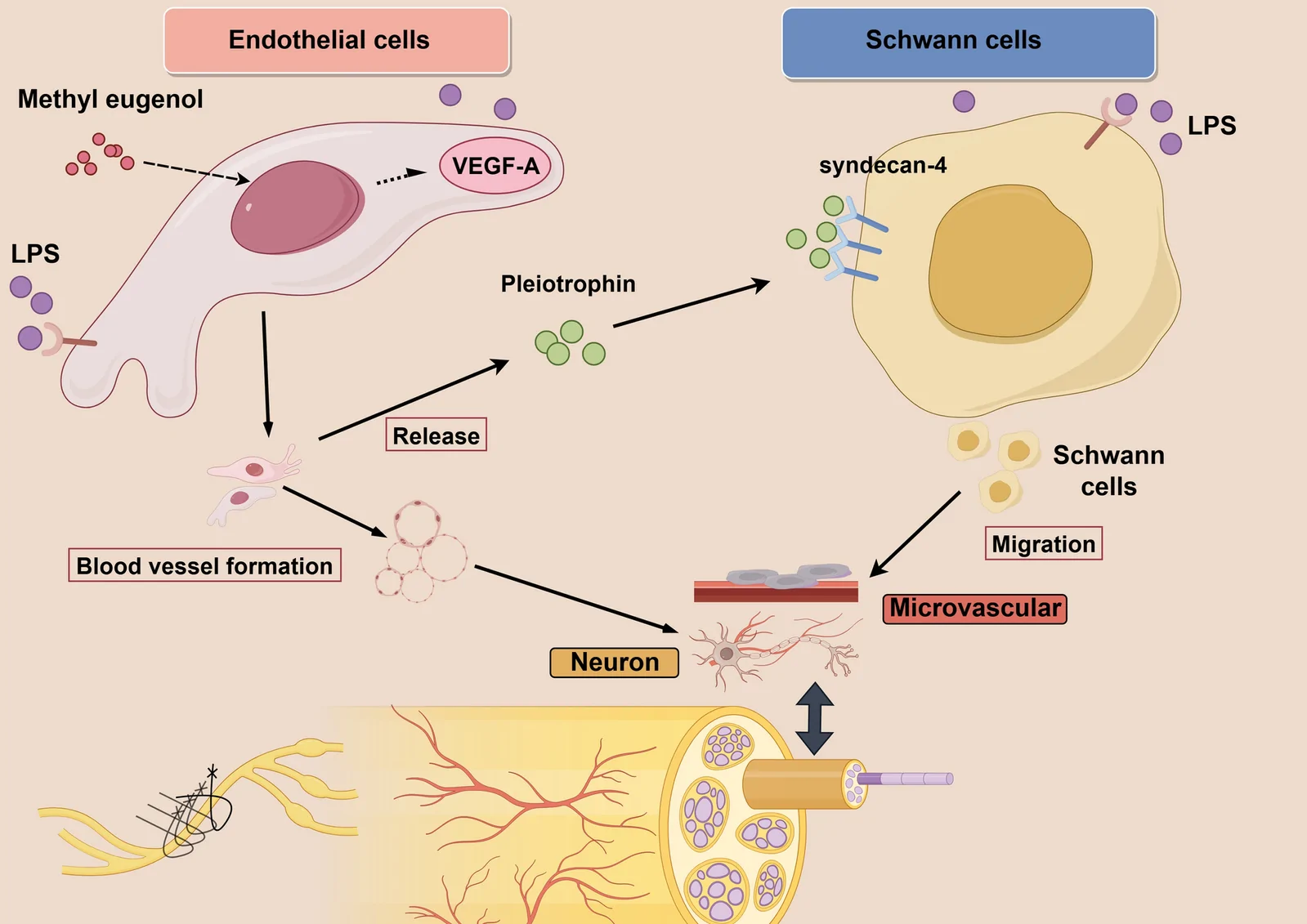

## Highlights

1. Joint analysis of single-cell sequencing and mRNA data revealed that nerve injury reduced communication between endothelial cells and Schwann cells.
2. Nerve injury led to neural microcirculatory disorders and neuroinflammation.
3. Methyl eugenol promoted RAOEC tube formation and increased the release of Ptn, which acted on Sdc4 in RSC96 cells to enhance migration.
4. In CCI rats, methyl eugenol treatment improved neural microcirculation and promoted nerve repair via the Ptn/Sdc4 pathway.

## Introduction

Sciatica describes radiating leg pain resulting from compression or inflammation of the lumbosacral nerve roots of the sciatic nerve. Sciatica occurs in 1–5% of individuals annually, and the lifetime prevalence of sciatica ranges from 13 to 40% [1]. The standard first-line treatment for sciatica involves a conservative approach that includes the use of conventional medications, such as nonsteroidal anti-inflammatory drugs (NSAIDs) [2–4].

However, such treatment offers limited pain relief and is frequently associated with side effects such as dizziness, drowsiness, sedation, allergic reactions, and liver or gastrointestinal issues. Alternatively, invasive treatments such as surgery are also available; however, no significant difference in outcomes has been observed between pharmaceutical therapy and surgery after more than four years of follow-up [5].

Moreover, conservative treatments seem to offer no greater benefit than placebo for the management of sciatica [2]. Therefore, new drugs for the treatment of sciatica are urgently needed.

Neural injury can lead to sciatica, and our previous studies revealed that promoting nerve repair can help alleviate sciatica [6, 7]. In long-gap peripheral nerve injuries, Schwann cell transplantation enhances both structural and functional recovery [8]. Autologous Schwann cell transplantation has been shown to promote the repair of sciatic nerve injuries with 7.5 cm and 5 cm gaps [9]. Schwann cell migration is directed by vascular endothelial cells during peripheral nerve repair, emphasizing the close connection between angiogenesis and nerve regeneration [10]. Furthermore, complex molecular interactions that regulate both peripheral nerve repair and angiogenesis have been demonstrated [11]. Vascular reconstruction plays a crucial role in nerve regeneration. However, the link between improved nerve microcirculation and enhanced nerve repair remains unclear. In this study, we found that communication between blood vessels and Schwann cells, particularly via the pleiotrophin/syndecan-4 signalling axis, was diminished after chronic constriction injury (CCI). Pleiotrophin (Ptn), a growth factor highly expressed in the nervous system, promotes neuronal survival, neurite outgrowth, and cell migration [12]. Syndecan-4 (Sdc4), which is highly abundant in the Schwann cells of the sciatic nerve, plays a key role in nerve function, regulates neuronal development, modulates cell migration and remyelination, and facilitates nerve repair [13].

*Asarum heterotropoides* F. Schmidt has been widely used as a remedy for inflammatory diseases, toothache, headache, local anesthesia, and other pains [14]. Methyl eugenol is identified as a key bioactive compound of *Asarum* that can be useful for distinguishing different Asarum species [15]. Methyl eugenol shows promise as a potent local anaesthetic and pain reliever [16], which are consistent with the traditional medicinal uses of *Asarum,* but its effects on nerve repair are unclear. Through mRNA sequencing and single-cell sequencing analysis, this study reveals that Ptn/Sdc4 participates in communication between endothelial cells and Schwann cells. We therefore investigated the interactions between endothelial cells and Schwann cells and found that methyl eugenol enhanced these interactions by increasing IL34/CSF1R signalling. Additionally, methyl eugenol promoted nerve repair in rats subjected to CCI.

## Methods and materials

### Subjects

Sixty-four male Sprague–Dawley rats (8–9 weeks old, weighing 200–250 g) were purchased from Guangzhou Weitong Lihua Biological Co., Ltd. The rats were housed in groups of five per cage at the Animal Experimental Center of the Medical College of Jinan University on a 12-h light/dark cycle under specific pathogen-free (SPF) conditions. Ethical approval (No. IACUC-20220314-12) was granted on March 14, 2022. All animal experiments were conducted in accordance with the National Institutes of Health Guide for the Care and Use of Laboratory Animals and were approved by the Animal Ethics Committee of Jinan University. All animals were uniquely identified by ear tags and randomly allocated to experimental groups using R (R Foundation for Statistical Computing, Vienna, Austria).

### Reagents

Ibuprofen (H20066) was obtained from the First Affiliated Hospital of Jinan University. The antibodies used in this study included: anti-Syndecan-4 (AF0831, Affinity), anti-PTN (DF7200, Affinity), anti-β-actin (AF7018, Affinity), anti-Caspase-3 (380189, Zenbio), anti-iNOS (ER1706-89, Huabio), anti-nNOS (CPA5524, Cohesionbio), anti-VEGF (PTM5278, PTMab), anti-IL-1β (CPA1586, Cohesionbio), and anti-COX-2 (33345, SAB). ELISA kits for IL-1β (MM-0047R1), IL-6 (MM-0190R1), TNF-α (MM-0180R1), TGF-β (MM-20594R1), IL-8 (MM-0175R1), and COX-2 (MM-70679R1) were purchased from Jiangsu Meimian Industrial Co., Ltd. (China). Methyl eugenol (D0510790250, 98%) was procured from Energy-Chemical Chemistry, and its SMILES is as follows: COC1=C(C=C(C=C1)CC=C)OC, as shown in Fig. 4A.

### Bioinformatics analysis of single-cell sequencing and mRNA sequencing data

mRNA sequencing data for the rat sciatic nerve were acquired from our previous publication [7]. Gene expression analysis was performed using DESeq2, with thresholds of |log_2_FC|≥ 0.5 and *P* < 0.05. Differentially expressed genes (DEGs) were subsequently subjected to Kyoto Encyclopedia of Genes and Genomes (KEGG) pathway analysis and gene set enrichment analysis (GSEA) using the clusterProfiler R package.

Single-cell sequencing data for the rat sciatic nerve (GSE146958) [17] were utilized. Data processing and analysis were performed using R (version 4.3) and Seurat (version 4.4). Cells with 200–4000 detected genes and < 20% mitochondrial gene content were retained for downstream analysis. After data integration, the Harmony algorithm was applied to correct for batch effects. Dimensionality reduction and clustering were conducted using dimensions 1–30 with a resolution of 0.8, resulting in the identification of 18 distinct cell types [18]. Cell clusters were visualized using uniform manifold approximation and projection (UMAP) and t-distributed stochastic neighbor embedding (t-SNE). Cell types were annotated based on known marker genes (Table 1). Finally, cell–cell interactions in the sciatic nerve were analyzed using the CellChat R package. Differential ligand–receptor interactions between conditions/groups were identified using CellChat’s built-in statistical framework with permutation testing (default CellChat procedure). Interactions were considered significantly differential at *P* < 0.05 (multiple testing correction by Benjamini–Hochberg FDR, with FDR < 0.05 used where indicated).

**Table 1:** Cell markers

Cell types	Marker
Fibroblasts	Pi16
Connective tissue	Abca6
Macrophages	Ctss
Mesenchymal	Clec11a
Schwann cells	Sostdc1
Endothelial cells	Rbp7
Smooth muscle cells	Myh11
T memory cells	Top2a
NK cells	Ms4a4b
Epithelial cells	Slc16a1
T cells	Trem1
Neutrophils	Mrgprb1

### Cell cultivation

Rat Schwann cells (RSC96 cells) and rat aortic endothelial cells (RAOECs) were cultured in DMEM supplemented with 10% fetal bovine serum and 1% penicillin–streptomycin in an incubator at 37 °C with 5% CO_2_. Cells were subcultured 2–3 times per week and used for experiments at passages 8–10.

Cell viability was assessed using the Cell Counting Kit-8 (CCK-8) assay. After enzymatic digestion, cells were resuspended in complete culture medium. A 200 μL cell suspension containing 3 × 10^3^ cells was seeded into 96-well plates, including blank wells (containing culture medium only) and untreated control wells. After 24 h of incubation under standard culture conditions, cells were treated with 200 μL of medium containing methyl eugenol at various concentrations (10, 20, 30, 40, 50, 60, 70, and 80 μM), with six replicate wells per concentration.

### RNA interference of Ptn and Sdc4 in RAOECs and RSC96 cells

Small interfering RNA (siRNA) sequences targeting Ptn or Sdc4 (Ptn siRNA and Sdc4 siRNA) were designed and synthesized by Tsingke Biotechnology Co., Ltd. The siRNA sequences are listed in Table 2. Ptn siRNA was transfected into RAOECs to knock down Ptn expression, whereas Sdc4 siRNA was transfected into RSC96 cells to reduce Sdc4 expression. Transfection efficiency was evaluated by Western blotting.

**Table 2:** siRNA sequences

Names	Sequence(5′–3′) sense	Sequence(5′–3′) antisense
Ptn siRNA	GAUGUAAGAUCCCUUGCAA	UUGCAAGGGAUCUUACAUC
Sdc4 siRNA	UGACGAUGUGUCCAACAAA	UUUGUUGGACACAUCGUCA

### Preparation of RAOEC-conditioned medium

RAOECs were divided into five groups: (A) control, (B) LPS (1 µg/mL), (C) LPS + methyl eugenol (20 µM), (D) LPS + Ptn siRNA (50 nM), and (E) LPS + methyl eugenol (20 µM) + Ptn siRNA (50 nM).

To prepare RAOEC-conditioned medium, 1.0 × 10^6^ RAOECs were seeded into 100-mm culture dishes and allowed to adhere overnight. The culture medium was then replaced with 5 mL of serum-free medium, and the corresponding treatments were applied according to group assignment (LPS at a final concentration of 1 µg/mL, methyl eugenol at 20 µM, and Ptn siRNA at 50 nM). After 24 h of treatment, the supernatant was collected, centrifuged at 1000 × *g* for 5 min, and subsequently filtered through a 0.22-µm filter. The resulting filtrate was used as RAOEC-conditioned medium for subsequent experiments.

### Experimental grouping of RSC96 cells

RSC96 cells were seeded into six-well plates and allowed to attach overnight. The cells were prepared under two conditions: untreated (control) and transfected with Sdc4 siRNA at a final concentration of 50 nM. Under each condition, RSC96 cells were then exposed to RAOEC-conditioned medium obtained from the different RAOEC treatment groups. The conditioned medium was mixed with fresh culture medium at a 1:1 ratio and applied to the cells for 24 h before subsequent experiments.

### Tube formation assay

Matrigel was thawed at 4 °C overnight. Prechilled 24-well plates and tools were prepared, and 30 μL of Matrigel was evenly spread in each well. To allow initial gelation, the plates were incubated at 4 °C overnight, followed by equilibration at 37 °C for 30 min in a humidified incubator to complete polymerization. RAOECs (1.5 × 10^5^ cells/well) suspended in 500 μL of conditioned medium were then seeded onto the polymerized Matrigel. Images of the vascular network were captured using an inverted light microscope at magnifications of 40× and 100× at 2 and 6 h, with three randomly selected fields per well. The number of branch points and the total segment length were quantified using ImageJ software.

### Wound healing assay

RSC96 cells and RAOECs were cultured in six-well plates at a density of 5 × 10^5^ cells per well in 2 mL of complete medium. After 24 h, when the cells reached confluence, reference lines were marked on the bottom of the plates using a laboratory marker. Three uniform linear wounds were created in each well using a sterile 200 μL pipette tip. The wells were then gently washed three times with phosphate-buffered saline (PBS, pH 7.4) to remove dislodged cells. Culture medium and drugs were subsequently added according to the experimental groups.

### Animal experiments

Sciatic nerve CCI was induced according to established protocols [6, 7, 19–22]. Following exposure of the right sciatic nerve, four 4-0 silk sutures were tied 1 mm apart proximal to the trifurcation. After surgery, penicillin was applied to the wound to prevent infection. In the sham group, the sciatic nerve was exposed but not ligated. In the first rat experiment, a total of 32 rats (n = 8 per group) were divided into four groups: the Sham, CCI 7 d, CCI 14 d, and CCI 21 d groups. In the second experiment, 32 rats were randomly assigned to four groups (n = 8 per group): (1) the Sham group, in which the sciatic nerve was exposed but not ligated and 0.9% saline (12 mL/kg) was administered intragastrically each day; (2) the CCI model group, in which the rats were subjected to CCI followed by daily intragastric administration of 0.9% saline at 12 mL/kg; (3) the ibuprofen group, in which the rats were subjected to CCI surgery followed by daily intragastric administration of ibuprofen (20 mg/kg); and (4) the methyl eugenol group, in which the rats were subjected to CCI surgery followed by daily intragastric administration of methyl eugenol (100 mg/kg) [23]. All the treatments were administered for 21 days.

### Von Frey tests and blood flow in the sciatic nerve

In accordance with methods described in previous studies, the von Frey test was used to evaluate mechanical hyperalgesia by determining the 50% mechanical withdrawal threshold (MWT) [7]. Testing began with a 2-g filament, and each calibrated filament (ranging from 2.0 to 26.0 g) was applied perpendicularly to the plantar surface of the right hind paw. A positive response was defined as rapid paw withdrawal, licking, or shaking within 2 s of stimulation. Upon detection of a positive response, the pain threshold was determined by the following formula: 50% MWT = 10^[Xf+kδ]/10,000^. Mechanical hyperalgesia of the right hind paw was evaluated on postoperative days 1, 4, 7, 14, and 21; three measurements were taken at 10-min intervals for the right hind paw, after which the mean values were calculated.

Following sciatic nerve exposure, blood flow was monitored for 120 s. The scan head was positioned approximately 20 cm above the sciatic nerve region. Images were acquired at a rate of one frame per second at normal resolution.

### Haematoxylin and eosin (H&E) staining, immunohistochemistry, and enzyme-linked immunosorbent assay (ELISA)

Liver, kidney, and sciatic nerve tissues were fixed in 4% paraformaldehyde, processed into paraffin-embedded sections (3 μm thick), and subjected to H&E staining. Following deparaffinization, the tissue sections were sequentially subjected to H&E staining according to standard protocols.

For immunohistochemical analysis, sciatic nerve sections were treated with sodium citrate antigen retrieval solution (1:1000 dilution, pH 6), followed by incubation with primary antibodies (1:200 dilution) and then the appropriate secondary antibody. The stained samples were then examined using an Olympus microscope.

To measure the concentrations of inflammatory factors in the sciatic nerve, the tissues were homogenized in extraction buffer. The subsequent steps were performed according to the manufacturer's guidelines, and the optical density was measured using a microplate reader.

### Western blot analysis

Total protein from the right sciatic nerve, RSC96 cells, and RAOECs was extracted in RIPA buffer followed by centrifugation (14,000× *g*, 15 min) [24–26]. Thirty micrograms of protein were separated on a 10–12% SDS-PAGE gel and then transferred onto a PVDF membrane. The membrane was blocked with 5% skim milk powder for 1 h, followed by incubation with primary antibodies (1:1000 dilution) at 4 °C for 12 h [27, 28]. The samples were then incubated with secondary antibody (1:10,000 dilution) for 1 h. The protein bands were visualized using a ChemiDoc XRS imager. Each experiment was performed in triplicate.

### Statistical analysis

Allocation concealment was ensured by having group assignments prepared by an independent researcher not involved in the subsequent experiments or data analysis. For behavioral testing, histological evaluations, and Western blot quantification, experimenters were blinded to the group assignments throughout data collection and analysis.

All data were analyzed using GraphPad Prism 9 and R version 4.0.1. Data are expressed as the mean ± standard deviation. Normality of data distribution was assessed using the Shapiro–Wilk test. Parametric tests were used for normally distributed data, whereas nonparametric tests were applied when normality was not met. Behavioral data were analyzed using repeated-measures ANOVA. The sequencing data analysis methods are described above. Other data, including ELISA data, PCR data, Western blot data, and immunohistochemical data, were analyzed using one-way ANOVA followed by Tukey’s multiple comparisons test. A *p* value < 0.05 was considered statistically significant.

## Results

### Single-cell sequencing and mRNA analysis of the sciatic nerve

**Fig. 1:**
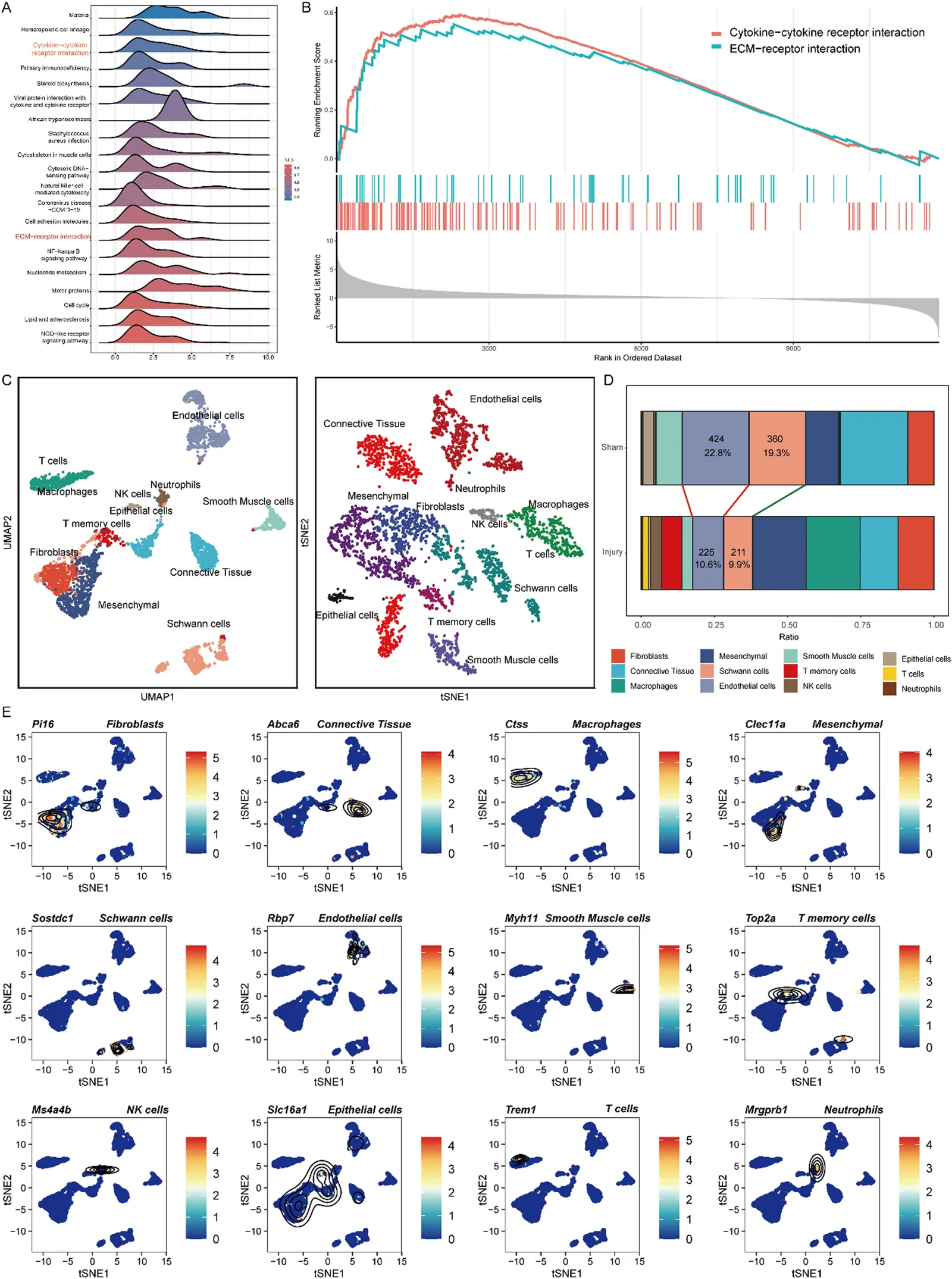
Transcriptome and single-cell profiling of the sciatic nerve. **A**, **B** KEGG and GSEA analyses of the cytokine–cytokine receptor interaction and ECM–receptor interaction pathways. **C** t-SNE/UMAP plots showing clustered cell populations. **D** Proportions of different cell types. **E** Representative marker genes for each cell cluster

KEGG and GSEA analyses revealed significant enrichment of the “ECM–receptor interaction” and “cytokine–cytokine receptor interaction” pathways following sciatic nerve injury (Fig. 1A, B).

Statistical analysis of cell proportions (Table 3) in t-SNE/UMAP-annotated cell clusters from the sciatic nerves of rats subjected to CCI showed that the Sham group contained greater proportions of endothelial cells (424; 22.8%) and Schwann cells (360; 19.3%). In contrast, the CCI group exhibited a marked reduction in the proportions of endothelial cells (225; 10.6%) and Schwann cells (211; 9.9%) (Fig. 1C, D). These results suggest that sciatic nerve injury is associated with increased macrophage infiltration and a concomitant decrease in Schwann and endothelial cell populations (Fig. 1C, D). The corresponding cell-type marker genes are shown (Fig. 1E).

**Table 3:** Cell numbers and proportions

Cell types	Injury(number)	Injury(proportion)	Sham(number)	Sham(proportion)
Fibroblasts	260	0.122	167	0.090
Connective tissue	277	0.130	429	0.231
Macrophages	392	0.184	9	0.005
Mesenchymal	389	0.183	211	0.113
Schwann cells	211	0.099	360	0.193
Endothelial cells	225	0.106	424	0.228
Smooth muscle cells	78	0.037	166	0.089
T memory cells	147	0.069	3	0.002
NK cells	81	0.038	13	0.007
Epithelial cells	15	0.007	67	0.036
T cells	43	0.020	1	0.001
Neutrophils	10	0.005	11	0.006

### Injury reduced communication between endothelial cells and Schwann cells

**Fig. 2:**
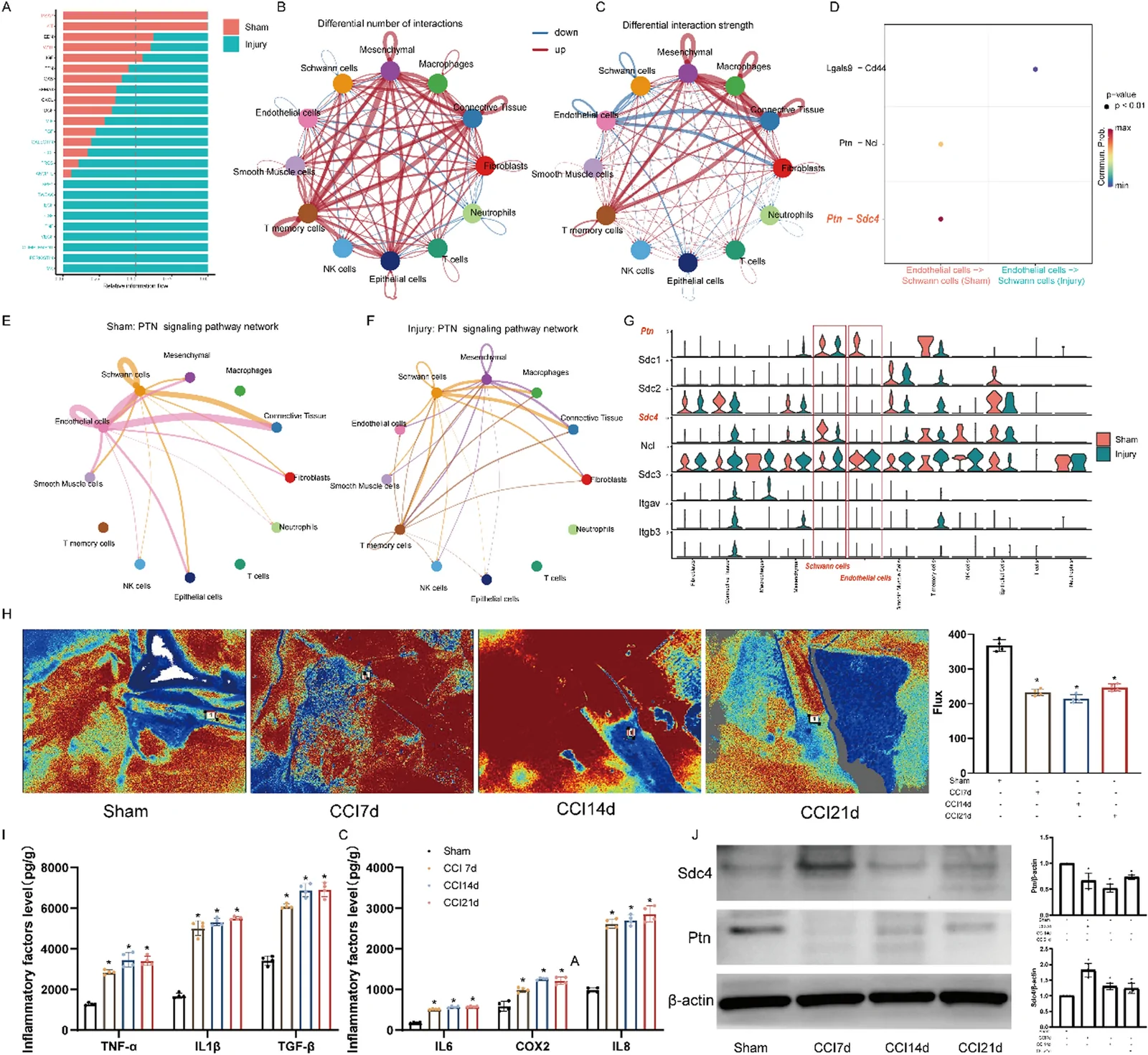
Cell–cell communication after sciatic nerve injury and experimental validation. **A**–**C** Cell–cell interaction proportions, numbers, and strengths. **D** Dot plot of ligand–receptor pairs between endothelial cells and Schwann cells. **E**, **F** Ptn signaling pathways in the Sham and CCI groups. **G** Violin plots showing Ptn and Sdc4 expression levels in the Sham and CCI groups. **H** Blood flow in the right sciatic nerve of CCI model rats (n = 4). **I** Levels of inflammatory factors (n = 4). **J** Western blot analysis of Sdc4 and Ptn expression (n = 3). *, compared with the Sham group; *P* < 0.05

To further investigate cell–cell interactions after sciatic nerve injury, we constructed a cell communication network (Fig. 2A). The cell–cell interaction number and strength plots revealed that interactions between Schwann cells and endothelial cells were reduced after sciatic nerve injury (Fig. 2B, C). Analysis of cell communication between endothelial cells and Schwann cells identified two key signaling pathways, Ptn/Sdc4 and Ptn/Ncl, with the interaction score of Ptn/Sdc4 exceeding that of Ptn/Ncl (Fig. 2D–F). Endothelial cells in the Sham group exhibited higher Ptn expression, which was reduced following sciatic nerve injury (Fig. 2G).

### CCI exacerbated microcirculatory disturbances in the sciatic nerve

The microcirculation in the sciatic nerve was reduced at postoperative day 7, 14, and 21 after CCI (Fig. 2H, *P* < 0.05). ELISA results showed increased TNFα, IL-1β, TGFβ, IL-6, COX-2, and IL-8 in the sciatic nerve of the CCI groups compared with the Sham group at postoperative days 7, 14, and 21 (Fig. 2I, *P* < 0.05). CCI also increased Sdc4 levels and reduced Ptn expression (Fig. 2J, *P* < 0.05).

### CCI increased the levels of VEGF-A, Sdc4, IL-1β, COX-2, iNOS, and nNOS

**Fig. 3:**
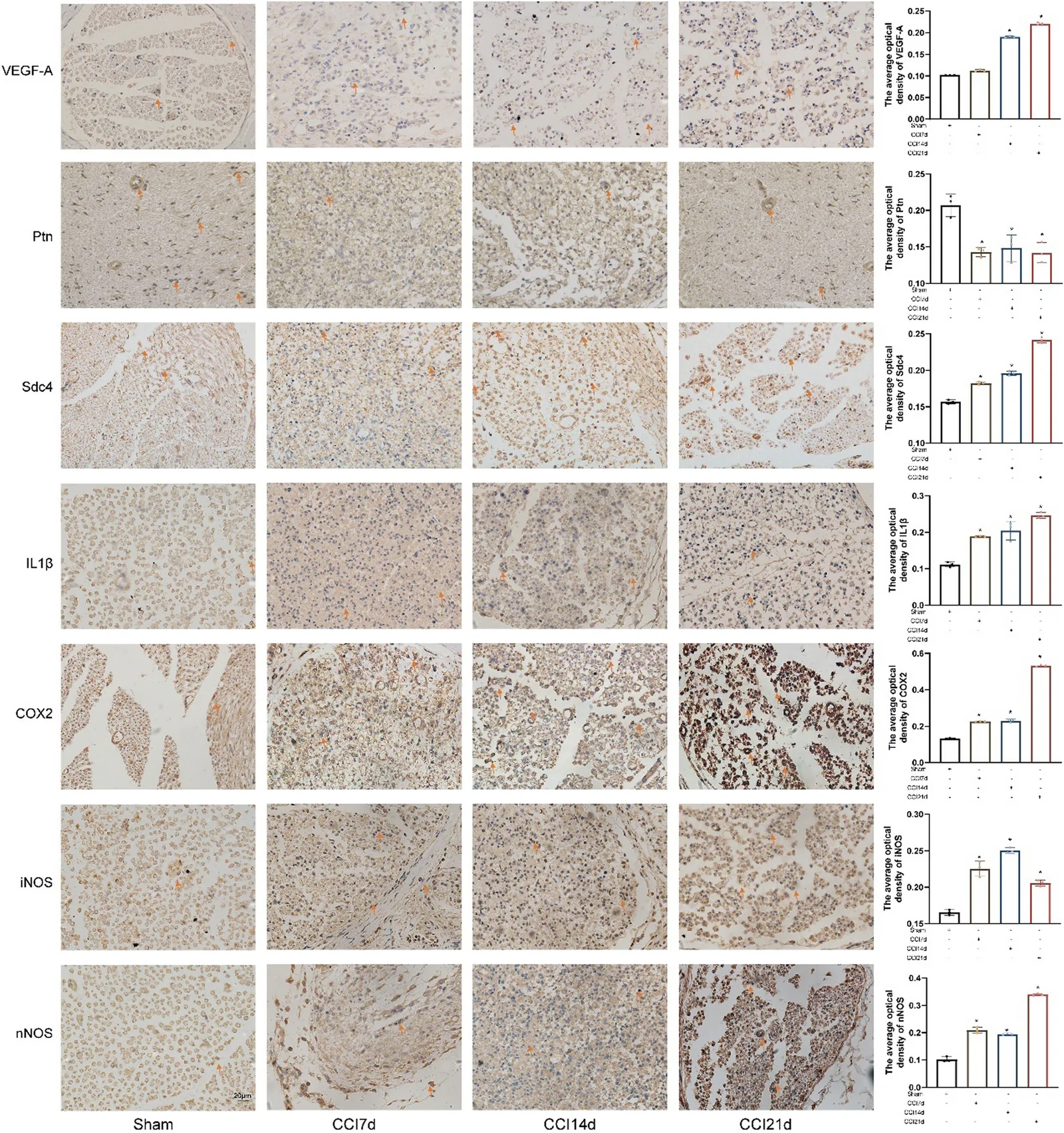
Immunohistochemical staining of VEGF-A, Ptn, Sdc4, IL-1β, COX-2, iNOS, and nNOS in the sciatic nerve (n = 3). *, compared with the Sham group;

Compared with the Sham group, the CCI groups exhibited higher levels of VEGF-A, Sdc4, IL-1β, COX-2, iNOS, and nNOS, at postoperative days 7, 14, and 21 after CCI (Fig. 3, *P* < 0.05). In contrast, Ptn expression was reduced after injury (Fig. 3, *P* < 0.05).

### Methyl eugenol promoted angiogenesis and Ptn secretion in RAOECs

**Fig. 4:**
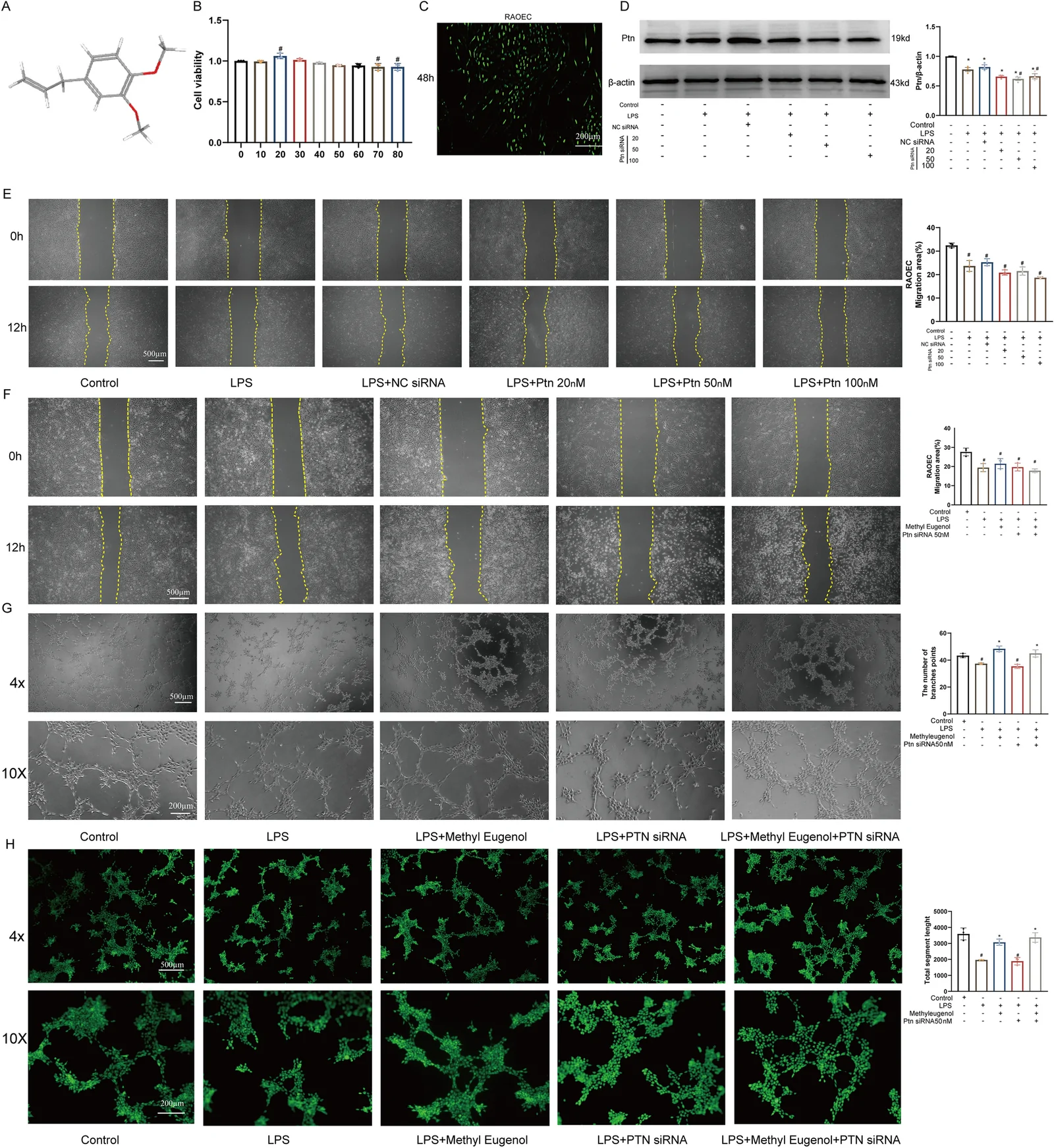
Effects of methyl eugenol on RAOECs. **A** Chemical structure of methyl eugenol. **B** CCK-8 assayshowing RAOEC viability (n = 3). **C** Fluorescence images of Ptn siRNA-transfected RAOECs (n = 3). **D** Western blot analysis of Ptn expression in RAOECs (n = 3). **E**, **F** RAOEC wound healing assay (n = 3). **G**, **H** Bright-field (G) and fluorescence (H) images of the RAOEC tube formation assay (n = 3). #, compared with the sham group; *, compared with the LPS group; *P* < 0.05

The chemical structure of methyl eugenol is shown (Fig. 4A). Methyl eugenol at 20 μM significantly increased cell viability (Fig. 4B, *P* < 0.05). Ptn siRNA (50 nM) reduced Ptn levels in RAOECs (Fig. 4C, D). Neither Ptn siRNA nor methyl eugenol inhibited RAOEC migration (Fig. 4E, F). In contrast, methyl eugenol treatment markedly increased the number of branch points and the total segment length of RAOECs (Fig. 4G, H, *P* < 0.05), indicating enhanced vascular network formation. Conversely, Ptn siRNA decreased these angiogenic parameters (Fig. 4G, H, *P* < 0.05).

### Ptn promoted Schwann cell migration

**Fig. 5:**
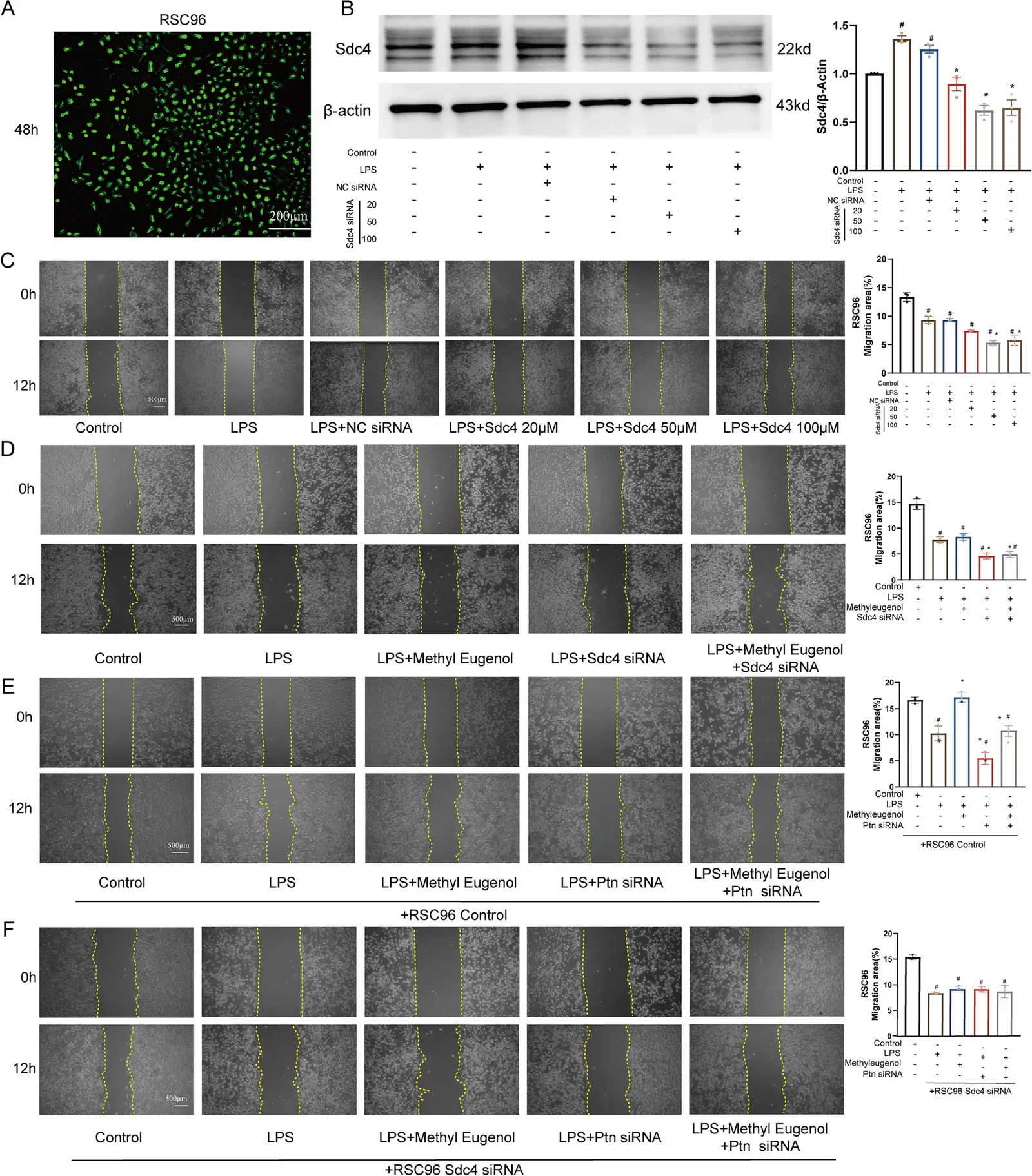
Effects of RAOEC-conditioned medium on RSC96 cells. **A** Fluorescence images of Sdc4 siRNA-transfected RSC96 cells (n = 3). **B** Western blot analysis of Sdc4 expression in RSC96 cells treated with 20 nM, 50 nM, or 100 nM Sdc4 siRNA (n = 3). **C**, **D** Wound healing assay of RSC96 cells treated with Sdc4 siRNA at 20 nM, 50 nM, or 100 nM, with or without methyl eugenol (n = 3). **E**, **F** Wound healing assay of RSC96 cells treated with RAOEC-conditioned medium (n = 3). #, compared with the Sham group; *, compared with the LPS group (*P* < 0.05)

Sdc4 siRNA (50 nM) effectively reduced Sdc4 expression levels (Fig. 5A, B). Scratch assays showed that Sdc4 siRNA significantly inhibited RSC96 cell migration (Fig. 5C, *P* < 0.05). Methyl eugenol treatment did not enhance RSC96 cell migration (Fig. 5D). To further validate these findings, RSC96 cells exposed to RAOEC-conditioned medium exhibited increased migration in the control group, whereas Sdc4 siRNA-transfected cells showed no significant change (Fig. 5E, F, *P* < 0.05). Furthermore, conditioned medium from methyl eugenol-treated RAOECs increased RSC96 cell migration, whereas Ptn siRNA or Sdc4 siRNA reversed this effect (Fig. 5E, F, *P* < 0.05).

### Methyl eugenol promoted neural microcirculation to relieve pain

**Fig. 6:**
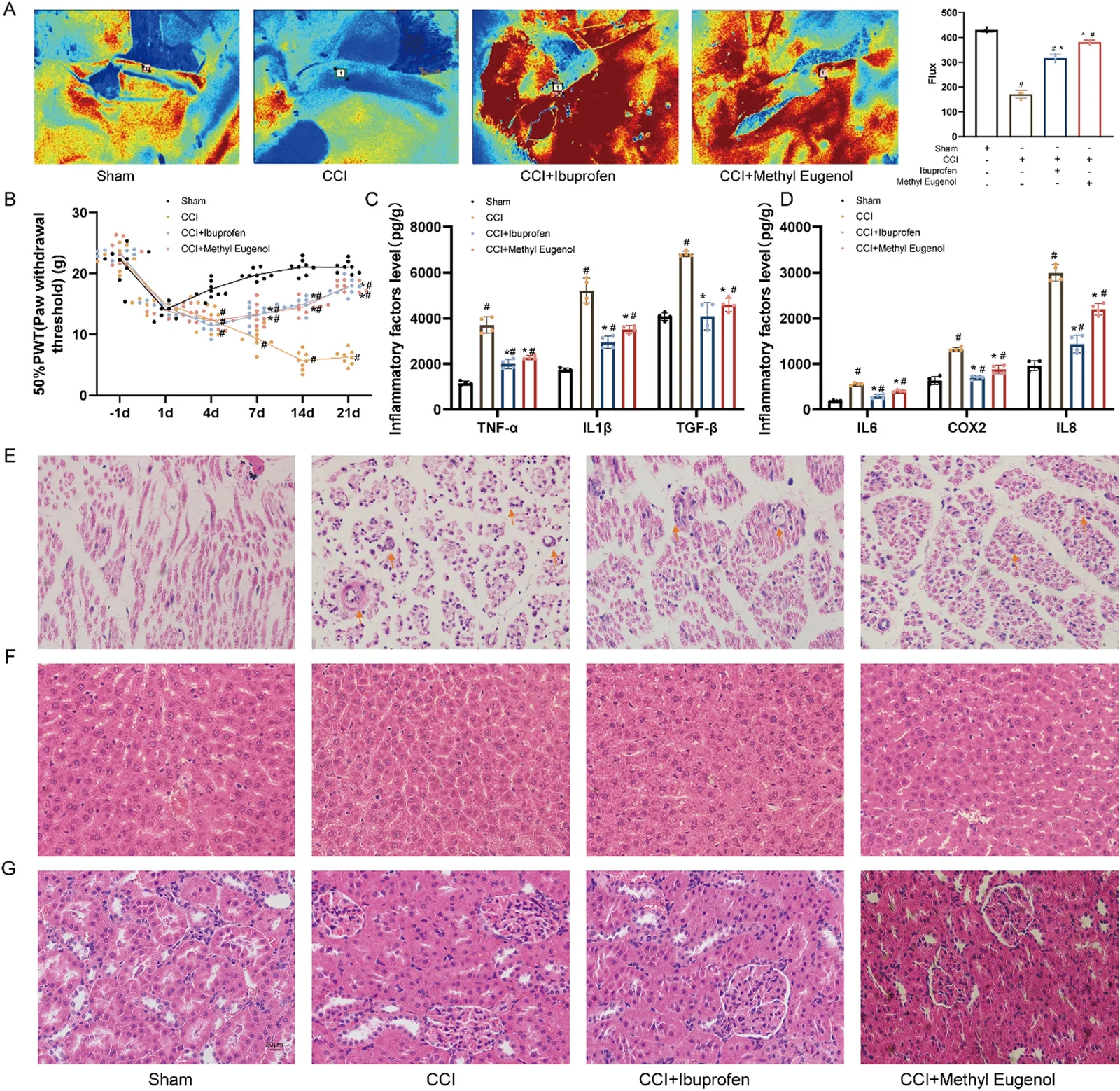
Effects of methyl eugenol on neural microcirculation and nerve repair. **A** Blood flow of the sciatic nerve (n = 4). **B** Von Frey test results (n = 8). **C**, **D** ELISA quantification of TNF-α, IL-1β, TGF-β, IL-6, COX-2, and IL-8 levels in the sciatic nerve (n = 4). **E**–**G** H&E staining of the sciatic nerve (**E**), liver (**F**), and kidney (**G**) (n = 3). #, compared with the Sham group; *, compared with the CCI group (*P* < 0.05)

Blood flow analysis revealed that CCI reduced sciatic neural microcirculation, whereas methyl eugenol treatment increased blood flow (Fig. 6A, *P* < 0.05). Methyl eugenol alleviated mechanical allodynia (Fig. 6B, *P* < 0.05). CCI induced upregulation of inflammatory mediators, including TNF-α, IL-1β, TGF-β, IL-6, COX-2, and IL-8, in the sciatic nerve, whereas methyl eugenol effectively suppressed this inflammatory response (Fig. 6C, D, *P* < 0.05).

H&E staining revealed that normal sciatic nerves exhibited well-organized axonal architecture with no signs of oedema or inflammatory infiltration. In contrast, CCI-induced injury caused severe structural disruption of the sciatic nerve, characterized by a disorganized architecture, oedema, and inflammatory cell infiltration (Fig. 6E). Methyl eugenol treatment significantly ameliorated these pathological changes, restored neural tissue integrity, and reduced inflammation (Fig. 6E). Additionally, H&E staining of the liver and kidneys revealed no significant histopathological alterations in either treatment group, indicating a favorable safety profile for both agents (Fig. 6F, G).

### Western blot analysis and immunohistochemical staining after methyl eugenol treatment

**Fig. 7:**
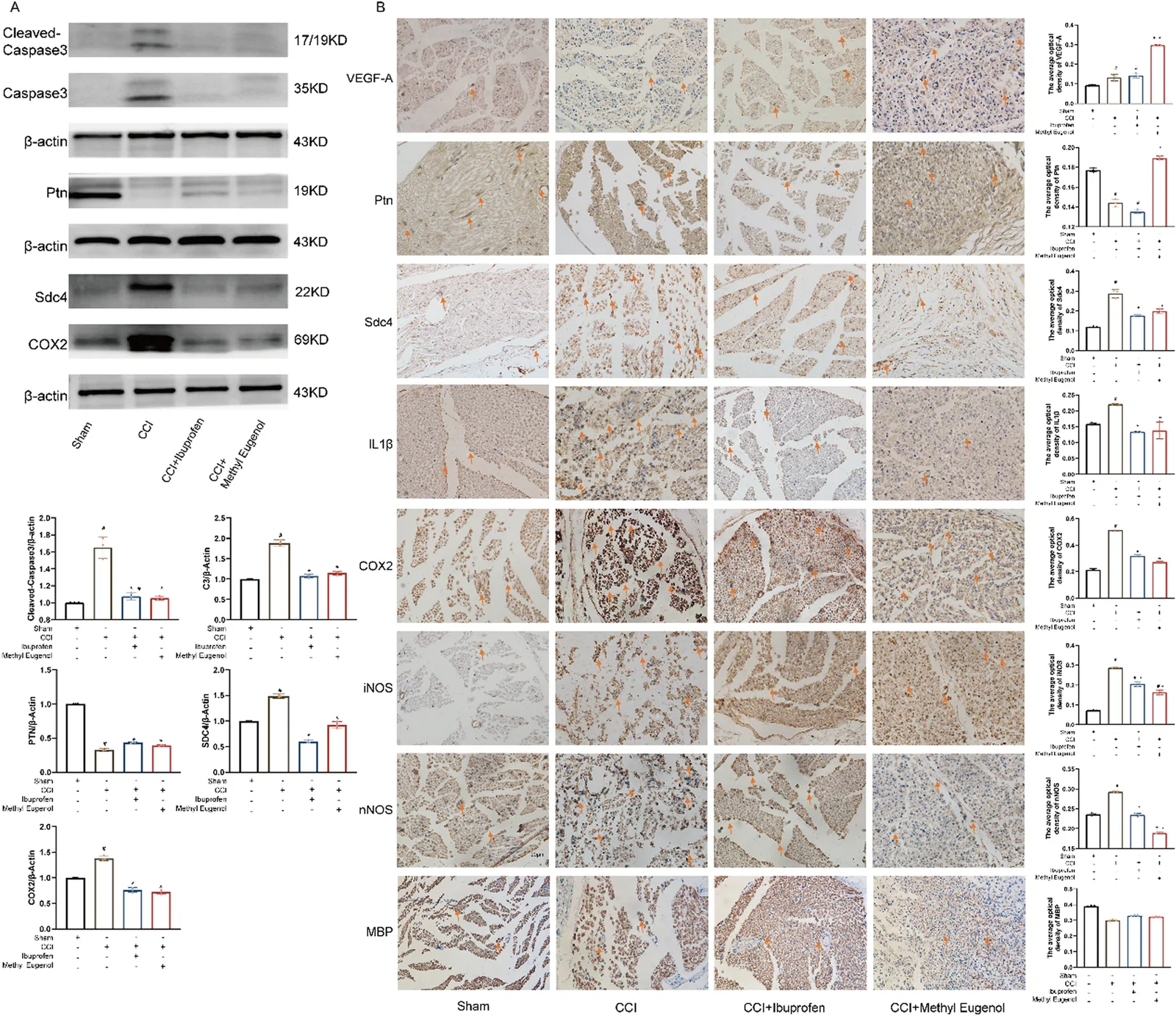
Western blot and immunohistochemistry results after methyl eugenol treatment. **A** Western blot analysis (n = 3). **B** Immunohistochemical staining (n = 3). #, compared with the Sham group; *, compared with the CCI group (*p* < 0.05)

Western blot analysis revealed that compared with the Sham group, the CCI group exhibited significantly increased levels of cleaved caspase-3, caspase-3, and COX-2 (Fig. 7A, *P* < 0.05). Immunohistochemistry revealed elevated expression of iNOS, nNOS, IL-1β, and COX-2 in CCI group, together with reduced MBP immunoreactivity (Fig. 7B, *P* < 0.05). Following CCI, Ptn levels were reduced, whereas Sdc4 expression was increased (Fig. 7A, B, *P* < 0.05). Treatment with ibuprofen and methyl eugenol significantly reduced the levels of cleaved caspase-3, caspase-3, and COX-2 (Fig. 7A, *P* < 0.05) and downregulated iNOS, nNOS, IL-1β, and COX-2 expression, while increasing MBP immunoreactivity (Fig. 7B, *P* < 0.05). Moreover, Ptn levels increased and Sdc4 expression decreased after treatment (Fig. 7A, B, *P* < 0.05).

### Double immunofluorescence staining of Ptn and Sdc4 in the sciatic nerve

**Fig. 8:**
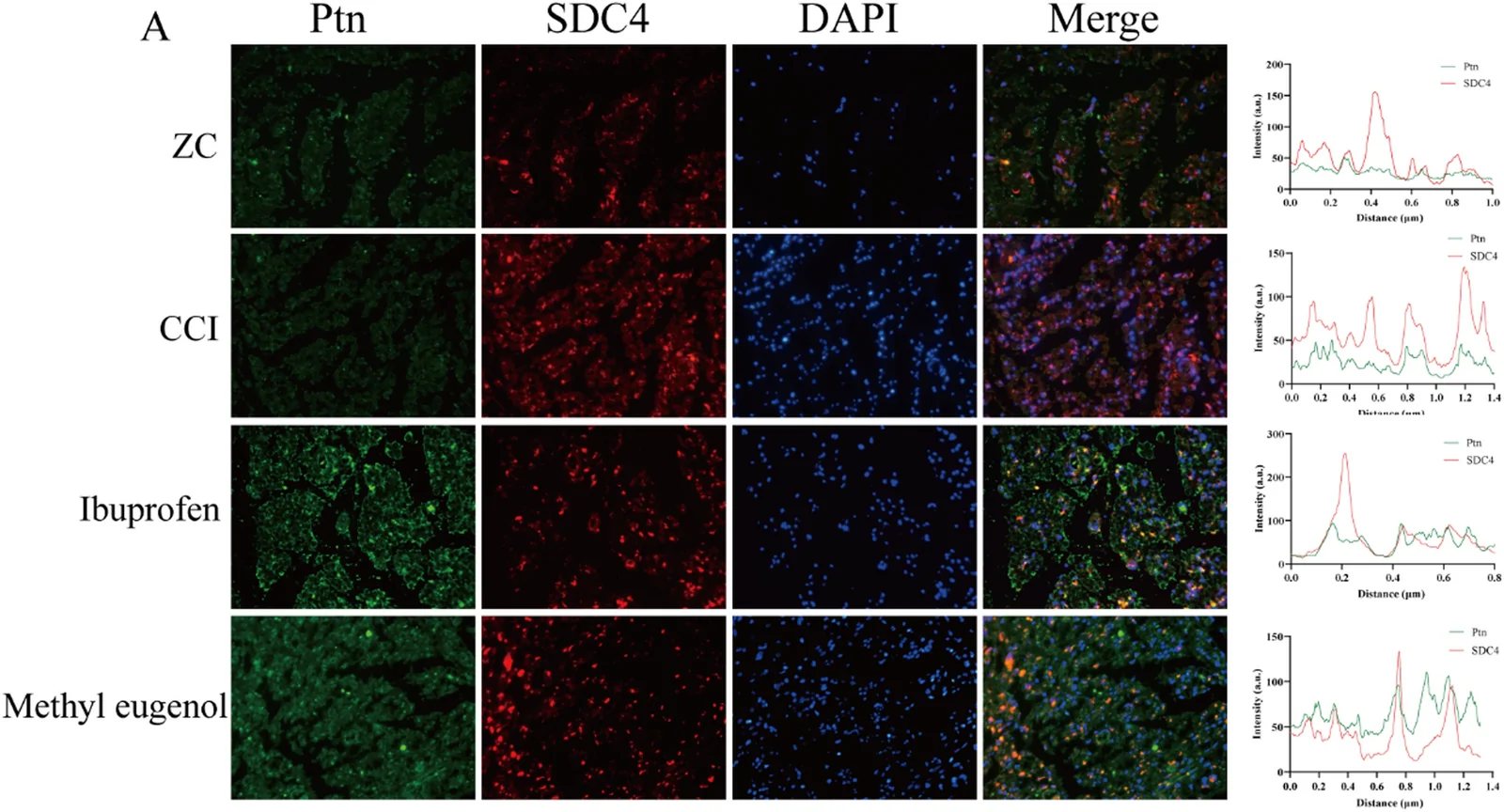
Double immunofluorescence staining and colocalization analysis of Ptn (green) and Sdc4 (red) in the sciatic nerve

Double immunofluorescence staining was performed to further evaluate the spatial expression patterns of Ptn (green) and Sdc4 (red) in the sciatic nerve (Fig. 8A). In the Sham group, Ptn and Sdc4 signals were moderately expressed and partially overlapped. In contrast, the CCI group exhibited decreased Ptn fluorescence intensity and increased Sdc4 expression, accompanied by reduced spatial overlap in the merged images.

Following treatment, methyl eugenol markedly increased Ptn fluorescence intensity and reduced Sdc4 expression compared with the CCI group, whereas ibuprofen showed a moderate effect. Line profile analysis further demonstrated an imbalance between Ptn and Sdc4 signals after CCI, which was partially restored by methyl eugenol treatment. These findings are consistent with the disruption and subsequent recovery of Ptn/Sdc4-mediated endothelial–Schwann cell communication.

## Discussion

In this study, the results suggest that methyl eugenol effectively improved neural microcirculation and promoted nerve repair by regulating endothelial cell–Schwann cell (EC–SC) communication via the Ptn/Sdc4 pathway. The main conclusions of this study are summarized as follows: (1) Joint analysis of single-cell sequencing and mRNA data revealed that nerve injury reduced communication between endothelial cells and Schwann cells, particularly via the Ptn/Sdc4 pathway; (2) nerve injury led to neural microcirculatory dysfunction and neuroinflammation; (3) methyl eugenol promoted RAOEC tube formation and increased Ptn release, which acted on Sdc4 in RSC96 cells to enhance cell migration; and (4) in rats subjected to CCI, methyl eugenol treatment improved neural microcirculation and promoted nerve repair via the Ptn/Sdc4 pathway.

Revascularization is essential for successful nerve repair [29], and the use of vascularized grafts has emerged as a promising strategy to address extensive peripheral nerve defects [30]. An injectable hydrogel containing VEGF-mimetic peptide nanoliposomes was shown to stimulate human umbilical vein endothelial cell (HUVEC) proliferation, migration, and angiogenesis in vitro through VEGF pathway activation; this hydrogel was also shown to enhance nerve regeneration in vivo via vascular remodelling and macrophage M2 polarization [31]. Additionally, VEGF-functionalized artificial nerve conduits have been shown to enhance neovascularization during axonal regeneration [32]. In this study, CCI led to reduced blood flow, whereas methyl eugenol treatment increased blood flow.

Schwann cell migration is vital for nerve repair, and Schwann cell transplantation has therapeutic potential through microenvironmental reconstruction and peripheral nerve regeneration [33]. As demonstrated in our previous studies, the promotion of nerve repair can alleviate pain via inhibition of Schwann cell apoptosis [6, 7, 19, 20, 34–36]. After injury, Schwann cells become activated and shift from a mature myelinating or nonmyelinating state to a proliferative, repair-oriented phenotype [37]. Following dedifferentiation, Schwann cells form axon-guiding Büngner bands through proliferation and longitudinal extension, while secreting neurotrophic factors, cytokines, growth signals, and extracellular matrix molecules that promote neuronal survival and axonal regeneration [38]. After CCI, increased cell apoptosis was observed. Following methyl eugenol treatment, apoptosis was observed, which may be associated with improved nerve repair.

Nerves and blood vessels often run in parallel, and endothelial cells guide the formation of Büngner bands that support axonal regeneration [30]. However, the underlying mechanism remains unclear. Analysis of single-cell data revealed that the Ptn/Sdc4 pathway is crucial for communication between endothelial cells and Schwann cells. While methyl eugenol did not promote RSC96 cell migration, it did stimulate RAOEC tube formation. Furthermore, methyl eugenol increased Ptn secretion from RAOECs, which subsequently acted on Sdc4 in RSC96 cells and led to increased cell migration. These findings indicate that methyl eugenol promotes RSC96 cell migration through an endothelial cell-dependent mechanism via the Ptn/Sdc4 pathway. Although RAOECs do not fully represent neural microvascular endothelial cells, they retain key endothelial characteristics relevant to angiogenesis and paracrine signaling, including growth factor secretion and ligand–receptor communication, which are central to the mechanisms examined in this study. Future studies using primary neural microvascular endothelial cells will be important to further confirm the physiological relevance of these findings.

With respect to drug toxicity, H&E staining of the liver and kidney revealed no obvious histopathological abnormalities. These findings indicate that the Ptn/Sdc4 pathway mediates Schwann cell–endothelial cell communication. Methyl eugenol augments this intercellular crosstalk and enhances neural microcirculation and nerve repair.

This study has certain limitations. Male rats were exclusively used to limit variability introduced by the estrous cycle and to improve data interpretability. However, sex-related differences in receptor expression, inflammatory responses, pain sensitivity, and drug metabolism may influence the efficacy of therapeutic interventions. Therefore, future studies including female animals are necessary to validate the efficacy of methyl eugenol and to assess potential sex-specific effects in sciatic nerve injury and repair.

In the CCI rat model, only a single dose level of methyl eugenol was evaluated for pharmacological validation. Dose–response relationships and optimal dosing regimens will be explored in future studies. In addition, ibuprofen was used as a positive control for pain and inflammation relief in the CCI model.

While our data in the rat CCI model indicate that methyl eugenol promotes neural microcirculation and nerve repair via endothelial–Schwann cell communication through the Ptn/Sdc4 axis, extrapolation to other species and humans requires caution. Pleiotrophin (Ptn) and syndecan-4 (Sdc4) are evolutionarily conserved, suggesting that the core ligand–receptor mechanism may be generalizable; however, species differences in endothelial and Schwann cell biology, drug metabolism, and immune responses can alter efficacy and safety. Validation using primary human cells (microvascular endothelium and human Schwann cells), alternative nerve injury models (crush, transection), inclusion of both sexes, aged animals, and comorbid conditions (e.g., diabetes) is needed, together with pharmacokinetic and toxicology studies, before clinical relevance can be established.

## Conclusion

CCI disrupts neural microcirculation and induces nerve injury. In cell co-culture experiments, methyl eugenol promoted RAOEC tube formation and increased the release of Ptn, acting on Sdc4 in RSC96 cells to enhance migration. In CCI rats, methyl eugenol improved neural microcirculationand enhanced nerve repair,potentially through modulation of endothelial–Schwann cell communication.

## Data Availability

The datasets supporting this study are accessible from the corresponding author upon reasonable request.

## Abbreviations

50% MWT: 50% Mechanical withdrawal threshold
CCI: Chronic constriction injury of the sciatic nerve
GSEA: Gene set enrichment analysis
KEGG: Kyoto Encyclopedia of Genes and Genomes
LPS: Lipopolysaccharide
NICE: National Institute for Health and Clinical Excellence
NSAIDs: Nonsteroidal anti-inflammatory drugs
PTN: Pleiotrophin
RAOECs: Rat aortic endothelial cells
RSC96 cells: Rat Schwann cells
Sdc4: Syndecan-4
